# Quantification of serum hepatitis B surface antigen in predicting the response of pegylated interferon alfa-2a in HBeAg-positive chronic hepatitis B with prior lamivudine exposure

**DOI:** 10.1186/1743-422X-10-277

**Published:** 2013-09-06

**Authors:** Min Weng, Wei-Zheng Zeng, Xiao-Ling Wu, Yong Zhang, Ming-De Jiang, Zhao Wang, De-Jiang Zhou, Xuan He

**Affiliations:** 1 Department of Digestion, General Hospital of Chengdu Military Region of PLA, Chengdu, Sichuan Province, People’s Republic of China

**Keywords:** Chronic hepatitis B, Lamivudine exposure, Pegylated interferon α-2a, Hepatitis B surface antigen quantification, Predictability

## Abstract

**Aims:**

Majority of previous studies of pegylated interferon α-2a (PegIFNα-2a) forced on naïve chronic hepatitis B (CHB) patients, and the data of PegIFNα-2a in therapy of patients with prior exposure to nucleos(t)ide analogues is rare. This study aimed to investigate the predictive role of serum quantitative hepatitis B surface antigen (HBsAg) in predicting sustained response of PegIFNα-2a in HBeAg-positive CHB patients with prior lamivudine exposure.

**Methods:**

Forty-six patients with prior lamivudine exposure received PegIFNα-2a for 12 months and followed-up for 6 months. The clinical features of responders and non-responders were compared, and the predictive role of quantitative HBsAg in predicting responders at the end of follow-up was evaluated. Responders were defined as an ALT normalization, HBeAg seroconversion and sustained virological response at the end of follow-up.

**Results:**

In this cohort, only 26.1% (12/46) patients were responders. The baseline characteristics of the responders and non-responders were similar; however, the rates of ALT normalization, HBV DNA undetectability and HBeAg seroconversion were all significantly higher in responders than that in non-responders. During the treatment and follow-up, the HBsAg levels were all significantly lower in responders than that in non-responders. In predicting reponders, the serum HBsAg cutoff of 6000 IU/mL at months 6 had a positive predictive value of 73.3 and a negative predictive value of 96.8%, and with an area under the receiver operating characteristic curve of 0.869.

**Conclusion:**

The responders toward PegIFNα-2a in CHB patients with prior lamivudine exposure is not high, and serum HBsAg <6000 IU/Ml at months 6 of on-treatment had a high value to predict long-term outcomes of treatment.

## Introduction

Hepatitis B virus (HBV) infection is a global public health problem [1]. It is estimated that a significant proportion of these patients will eventually die from complications (such as cirrhosis, liver failure and hepatocellular carcinoma) directly related to their chronic HBV infection, accounting for one million deaths annually [1]. In the last decade, with the introduction of nucleos(t)ide analogues (NAs) great strides have been made in the treatment of adult chronic hepatitis B (CHB) [2]. Though these oral NAs treatments may eliminate the HBV virus from the blood, they cannot clear intrahepatic covalently closed circular DNA (cccDNA) from a chronically infected liver, and do little to block the release of hepatitis B surface antigen (HBsAg) into the blood. As a result, majority of them only have a marginal effect on restoring the patients HBV immune response, and there are several problems naturally related to suboptimal response, viral resistance and the lack of a sustained curative response.

Previous studies had reported that the serum HBsAg level has some relationship with intrahepatic cccDNA [3], and serum HBsAg lower to an undetectable level may indicate that intrahepatic cccDNA is eradicated at all [4]. So currently, more and more scholars speculate that the main cause for the lack of a sustained curative response with existing oral NAs therapy may be that none of them targets the elimination of HBsAg from the blood, and therefor point that HBsAg quantitive measurement should be used as a benchmark for the efficacy evaluation of anti-viral treatment.

Besides NAs agents, interferon α (IFNα), especially its pegylated form, also has been approved and widely used in therapy of CHB in clinical practice [5]. And there are many evidence suggest that IFNs have two mechanisms of action: a direct antiviral effect achieved inhibiting the synthesis of viral DNA and by activating antiviral enzymes, and a second mechanism that increases the cellular immune response against hepatocytes infected with HBV. As compared to NAs, the advantages of IFNα therapy include a limited treatment course and less development of resistance, and even results in clinical cure, with HBsAg loss or seroconversion in a few patients. However, there are also a considerable proportion of patients responded poorly to IFNα therapy, and there are higher to 50% of hepatitis B e antigen (HBeAg) positive patients could not achieve HBeAg seroconversion [6]. Unfortunately, the exact molecular mechanism of this ineffectiveness of IFNα is still unknown.

Currently, how to identify and use indicators to predict treatment response has been widely concerned. In present study, we will evaluate the effectiveness of quantification of serum HBsAg in predicting the response of pegylated interferon α-2a in HBeAg-positive CHB patients with prior lamivudine exposure, and the findings of this study would provide an important reference for helping CHB patients achieve sustained curative response.

## Patients and methods

### Patients

HBeAg-positive chronic hepatitis B patients with prior lamivudine exposure and received pegylated interferon alfa-2a for recurring antiviral therapy were screened in this study, all of them were followed up at the Digestion Department of Chengdu Military General Hospital from January 2007 to December 2012. The inclusion criteria were as follows: adults (18–70 years), prior lamivudine exposure for more than 1 years, positive HBeAg statue, HBV DNA levels higher than 1.0×10^^5^ copies/mL, and elevated serum alanine amino-transferase (ALT) value. The exclusion criteria were as follows: ①co-infection with other hepatitis virus or human immunodeficiency virus; ②evidence of other causes of liver disease, such as autoimmune hepatitis and primary biliary cirrhosis; ③evidence of advanced liver diseases, such as decompensated cirrhosis, severe hepatitis, and hepatic carcinoma; ④poor compliance or no availability of detailed laboratory test results.

This study was carried out in accordance with the ethics committee of Chengdu Military General Hospital and informed consent was obtained from each participant.

### Study design and definition

This is a prospective observational study, and all eligible participants were administered pegylated interferon alfa-2a (PegINFα-2a) (Roche Pharmaceuticals, Shanghai, China) at a dose of 180 μg once a week by subcutaneous injection for 12 months. Quantification of serum HBsAg was carried out at baseline, months 3, 6 and at the end of treatment (12 months), and the quantitative HBV DNA and liver function was also assessed at each time-point.

According to the follow-up outcomes in this study, patients were designated as either responders or nonresponders. Responders were defined as an ALT normalization, accompanying with HBeAg seroconversion at the end of treatment and the presence of a sustained virological response. Patients who did not achieve the above-mentioned criteria were defined as NRs. And sustained virological response was defined as undetectable serum HBV DNA both at the end of therapy and 6-month of follow-up.

### Laboratory measurements

Liver function was studied using an automatic biochemical analyzer(Olympus AU5400, Olympus Corporation, Tokyo, Japan), serum HBV DNA was quantified by PCR assay with a lower limit of detection of 1000 copies/mL (DA AN GENE. Co., Ltd. Guangzhou, China), and serum HBsAg was measured quantitatively by a Roche chemiluminescence assay (Basel, Switzerland).

### Statistical analysis

Quantitative variables were expressed as mean and standard deviation. Categorical variables were presented as counts and percentages. The comparisons of quantitative variables were performed using T-test, and comparisons of qualitative variables were performed using Chi-square test or Fisher’s exact test as appropriate. The accuracy of serum HBsAg to predict response was assessed using the receiver operating characteristic (ROC) curve. All P-values were two-tailed. A *P*-value of less than 0.05 was considered statistically significant.

## Results

### Patient characteristics

A total of 59 patients were screened in this study and only 46 CHB patients (32 men and 14 women) were included. The other 13 patient were excluded because of poor compliance to treatment (N=6), prior lamivudine exposure less than 1 years(N=5), and HCV confection(N=2). Of patients included in this study, 26.1% (12/46) patients were responders and 73.9% (34/46) patients were non-responders towards PegIFN α-2a 12-month treatment, and their detailed demographic and clinical characteristics were shown in Table 1. There were no significant differences between responders and non-responders before PegIFN α-2a treatment when age, sex, baseline ALT level, viral load, and HBsAg levels were compared.

**Table 1:** Baseline characteristics of the responders and non-responders

**Variables**	**Responders**	**Non-responders**	***P*-value**
The number of patients (N,%)	12(26.1%)	34(73.9%)	
Age (years)	33.8±4.6	35.7±5.8	0.3113
Male (N,%)	8(66.7%)	24(70.6%)	1.0000
Body mass index (kg/m^2^)	22.6±2.1	22.1±1.9	0.4496
ALT (U/L)	168.5±54.2	131.7±67.9	0.0976
HBV DNA (log copies/mL)	6.1±1.3	6.4±0.5	0.2588
HBsAg (log IU/mL)	4.2±0.5	4.4±0.7	0.3686
Duration of prior LAM exposure (years)	1.5±0.6	1.7±0.5	0.2643

### The biochemical and virological changes between responders and nonresponders

At the end of 12-month PegINFα-2a treatment, both serum ALT and HBV DNA levels were lower than that at baseline for all patients. The subgroup comparison of serum ALT and HBV DNA levels between responders and nonresponders during the treatment are presented in Table 2. For responders, the ALT normalization rate was 41.7% (5/12), 66.7% (8/12) and 83.3% (10/12) at months 3, 6 and 12 respectively; and the HBV DNA undetectability rate was 50.0% (6/12), 83.3% (10/12), and 100% (12/12) at months 3, 6 and 12 respectively. For nonresponders, the ALT normalization rate was 44.1% (15/34), 61.7% (21/34) and 76.5% (26/34) at months 3, 6 and 12 respectively; and the HBV DNA undetectability rate was 35.3% (12/34), 47.1% (16/34), and 58.8% (20/34) at months 3, 6 and 12 respectively. In this cohort, the percentage of ALT normalization in responders was similar to that in nonresponders from months 3 to months 12, but statistically significantly at 6-month follow-up (100% versus 70.6%, *P*=0.0439). As compared to nonresponders, the undetectability rate of serum HBV DNA was significantly lower in responders since months 6 of treatment. In respect to HBeAg seroconversion, we also found that its rate either at months 12 of treatment (83.3% vs 32.4%, *P*=0.0055)or 6-month follow-up (100% vs 32.4%, *P*<0.0001) was significantly higher in responders than that in non-responders.

**Table 2:** Comparison of ALT, HBV DNA, HBeAg seroconversion and HBsAg during treatment and follow-up between the responders and nonresponders

**Variables**	**Rs**	**NRs**	***P*-value**
The number of patients	12	34	
ALT normalization (n,%)			
Months 3	5(41.7%)	15(44.1%)	1.0000
Months 6	8(66.7%)	21(61.7%)	1.0000
Months 12	10(83.3%)	26(76.5%)	1.0000
Six-month follow-up	12(100%)	24(70.6%)	0.0439
HBV DNA undetectability (n,%)			
Months 3	6(50.0%)	12(35.3%)	0.4949
Months 6	10(83.3%)	16(47.1%)	0.0430
Months 12	12(100%)	20(58.8%)	0.0088
Six-month follow-up	12(100%)	19(55.9%)	0.0042
HBeAg seroconversion			
Months 12	10(83.3%)	11(32.4%)	0.0055
Six-month follow-up	12(100%)	11(32.4%)	<0.0001
HBsAg (log IU/mL)			
Months 3	3.5±0.5	4.1±0.4	0.0001
Months 6	3.1±0.7	3.9±1.1	0.0235
Months 12	2.9±0.4	3.7±0.9	0.0049
Six-month follow-up	2.5±0.6	3.8±0.7	<0.0001

### Quantitative HBsAg change between responders and non-responders

As Table 2 showed that the mean HBsAg concentrations decreased consistently during treatment and remained at low levels during the post-treatment follow-up in responders. Conversely, HBsAg in nonresponders showed a relatively slight decrease during treatment and post-treatment follow-up. It was worth mentioning that there were 1 patient in responders obtained HBsAg loss, but anti-HBs statue was negative.

### The significance of HBsAg in predicting responders at six-month follow-up

Among 15 patients with HBsAg levels < 6000 IU/mL at months 6, 73.3% (11/15) were responders at the six-month of follow-up; among 31 patients with HBsAg levels ≥ 6000 IU/mL at months 6, 3.2% (1/31) were responder at the six-month of follow-up; and the difference between them was statistic significantly (*P*<0.0001). At months 6, the cutoff of 6000 IU/mL of HBsAg had a positive predictive value (PPV) of 73.3% and a negative predictive value (NPV) of 96.8% for predicting responders at the six-month of follow-up after PegIFN α-2a treatment, and the corresponding area under the ROC curve at months 6 were 0.869.

## Discussion

Lamivudine is the first anti-HBV agent approved in China, and it has been used in therapy of CHB patients for more than one decade. Thus, there are many CHB patients who have been treated with lamivudine, but the control of HBV DNA is not ideal because of the high rate of HBV resistance. Considering different mechanisms of anti-HBV and no cross-resistance to NAs, PegIFNα-2a also has been applied for salvage therapy of patient with resistance to lamivudine. Additionally, there was no data showed the existence of resistance to NAs could decrease the efficacy of interferon to HBV. In this study, though we found that 12-month PegIFNα-2a treatment resulted to 67.4% (31/46) undetectable HBV DNA, 78.3% (36/46) ALT normalization, and higher to 50% (23/46) HBeAg seroconversion, the combined response (ALT normalization combined with HBV DNA negativity and HBeAg seroconversion) was just 26.1%. So the salvage therapy of PegIFNα-2a for CHB patients with prior NAs exposure was not ideal, and how to optimize the existing treatment strategies and early predict long-term responses was necessary and important for the management of CHB.

In past decade, many evidence indicated that the intrahepatic cccDNA decreasing would be probably an ideal prognostic variable in predicting long-term outcomes of antiviral treatment [7], but it was still a research procedure, dependent on a liver biopsy, and hardly available to the practicing hepatologist. Though the use of quantitative HBV DNA is well established in monitoring antiviral effect of NAs and predicting long-term risk of hepatocellular carcinoma, its value in reflecting the immune control of HBV was extremely limited. Recently, quantification of serum HBsAg in naïve CHB patients has been recommended as an alternative marker for monitoring and evaluating efficacy of treatment [8-10]. And several independent studies have shown that the decline of serum HBsAg level during interferon treatment mimics that of intrahepatic cccDNA, suggesting that a decline or loss of serum HBsAg is correlated with a more effective immune control of HBV [3,4]. Moreover, the quantification of serum HBsAg has also been recommended as a useful index to guide interferon individualized treatment [11].

As we know, current therapeutic agents cannot completely remove HBV from liver; and the goal of antiviral treatment is just to slow down the progression of liver disease to cirrhosis and hepatocellular carcinoma, with the ultimate goal of improving survival. The HBsAg loss and eventual seroconversion would signify the best outcome possible for patients with CHB [9]. It has been reported that quantitative serum HBsAg and HBeAg are strong predictors of sustained HBeAg seroconversion to PegIFNa-2b in HBeAg-positive patients; and quantitative serum HBsAg level at 3 months of treatment could be used for the early prediction of a sustained response to PegIFN therapy in HBeAg-negative CHB patients [12]. In our study, among 12 responders with ALT normalization, HBV DNA negativity and HBeAg seroconversion, the serum HBsAg levels decreased consistently during treatment and remained at low levels during the post-treatment follow-up. Conversely, serum HBsAg in non-responders just showed a relative slight decrease during both treatment and post-treatment follow-up. Thus our findings further suggested that monitoring of serum HBsAg levels may predict ideal responses towards interferon treatment earlier. And this finding was also consistent with the findings of other published reports [5,12]. Additionally, we also found that the cutoff of 6000 IU/mL of serum HBsAg at months 6 had a PPV of 73.3% and an NPV of 96.8% for predicting combined responses of ALT normalization, HBV DNA negativity and HBeAg seroconversion.

In summary, the percentage of responders toward PegIFNα-2a in CHB patients with prior lamivudine exposure is not high; but the early decrease of serum HBsAg (< 6000 IU/mL at months 6) could be used as a predictor of sustained combined response. Due to the limitation of relatively small sample size, longer follow-up and larger sample size prospective trials should be required to confirm our findings.

## Competing interests

The contents are solely the responsibility of the authors.

## Authors’ contributions

Zeng WZ conceived the study and revised the manuscript critically for important intellectual content. Weng M, Wu XL and Zhang Y made substantial contributions to its design, acquisition, analysis and interpretation of data. Jiang MD, Wang Z, Zhou DJ and He X participated in the design, analysis and interpretation of data. All authors read and approved the final manuscript.

## References

[B1] Lai CL Ratziu V Yuen MF Poynard T Viral hepatitis B Lancet 2003 10 2089 2094 10.1016/S0140-6736(03)15108-2 14697813

[B2] Kumada T Toyoda H Tada T Kiriyama S Tanikawa M Hisanaga Y Kanamori A Niinomi T Yasuda S Andou Y Effect of nucleos(t)ide analogue therapy on hepatocarcinogenesis in chronic hepatitis B patients: a propensity score analysis J Hepatol 2013 10 427 433 10.1016/j.jhep.2012.10.025 23123221

[B3] Wong DK Seto WK Fung J Ip P Huang FY Lai CL Yuen MF Reduction of hepatitis B surface antigen and covalently closed circular DNA by nucleos(t)ide analogues of different potency Clin Gastroenterol Hepatol 2013 10 1004 1010 10.1016/j.cgh.2013.01.026 23376799

[B4] Zoulim F Testoni B Lebosse F Kinetics of intrahepatic cccDNA and serum HBsAg during antiviral therapy for chronic hepatitis B - lessons from experimental and clinical studies Clin Gastroenterol Hepatol 2013 10 1011 1013 10.1016/j.cgh.2013.04.010 23602824

[B5] Lampertico P Vigano M Colombo M Why do I treat HBeAg-negative chronic hepatitis B patients with pegylated interferon? Liver Int 2013 10 Suppl 1 157 163 23286860 10.1111/liv.12064

[B6] Lok AS McMahon BJ Chronic hepatitis B Hepatology 2007 10 507 539 10.1002/hep.21513 17256718

[B7] Cheng PN Liu WC Tsai HW Wu IC Chang TT Young KC Association of intrahepatic cccDNA reduction with the improvement of liver histology in chronic hepatitis B patients receiving oral antiviral agents J Med Virol 2011 10 602 607 10.1002/jmv.22014 21328373

[B8] Locarnini S Bowden S Hepatitis B surface antigen quantification: not what it seems on the surface Hepatology 2012 10 411 414 10.1002/hep.25732 22454331

[B9] Vigano M Lampertico P Clinical implications of HBsAg quantification in patients with chronic hepatitis B Saudi J Gastroenterol 2012 10 81 86 10.4103/1319-3767.93805 22421711 PMC3326981

[B10] Chen EQ Wang TT Bai L Tao CM Liang T Liu C Liao J Tang H Quantitative hepatitis B surface antigen titres in Chinese chronic hepatitis B patients over 4 years of entecavir treatment Antivir Ther 2013 doi:10.3851/IMP2579 [Epub ahead of print] 10.3851/IMP2579 23639885

[B11] Gheorghita VI Caruntu FA Curescu M Olaru I Radu MN Coltan G Streinu-Cercel A Use of quantitative serum HBsAg for optimization of therapy in chronic hepatitis B patients treated with pegylated interferon alfa-2a: a Romanian cohort study J Gastrointestin Liver Dis 2013 10 27 32 23539387

[B12] Peng CY Lai HC Li YF Su WP Chuang PH Kao JT Early serum HBsAg level as a strong predictor of sustained response to peginterferon alfa-2a in HBeAg-negative chronic hepatitis B Aliment Pharmacol Ther 2012 10 458 468 10.1111/j.1365-2036.2011.04973.x 22225574

